# Expansion of Myeloid Derived Suppressor Cells Contributes to Platelet Activation by L-Arginine Deprivation during SARS-CoV-2 Infection

**DOI:** 10.3390/cells10082111

**Published:** 2021-08-17

**Authors:** Alessandra Sacchi, Germana Grassi, Stefania Notari, Simona Gili, Veronica Bordoni, Eleonora Tartaglia, Rita Casetti, Eleonora Cimini, Davide Mariotti, Gabriele Garotto, Alessia Beccacece, Luisa Marchioni, Michele Bibas, Emanuele Nicastri, Giuseppe Ippolito, Chiara Agrati

**Affiliations:** 1 Department of Epidemiology, Pre-Clinical Research and Advanced Diagnostic, National Institute for Infectious Diseases “Lazzaro Spallanzani”-IRCCS, Via Portuense, 292-00149 Rome, Italy; germana.grassi@inmi.it (G.G.); stefania.notari@inmi.it (S.N.); gili.1581732@studenti.uniroma1.it (S.G.); veronica.bordoni@inmi.it (V.B.); eleonora.tartaglia@inmi.it (E.T.); rita.casetti@inmi.it (R.C.); eleonora.cimini@inmi.it (E.C.); davide.mariotti@inmi.it (D.M.); chiara.agrati@inmi.it (C.A.); 2 Clinical Department, National Institute for Infectious Diseases “Lazzaro Spallanzani”-IRCCS, Via Portuense, 292-00149 Rome, Italy; gabriele.garotto@inmi.it (G.G.); alessia.beccacece@inmi.it (A.B.); luisa.marchioni@inmi.it (L.M.); michele.bibas@inmi.it (M.B.); emanule.nicastri@inmi.it (E.N.); 3 National Institute for Infectious Diseases “Lazzaro Spallanzani”-IRCCS, Via Portuense, 292-00149 Rome, Italy; giuseppe.ippolito@inmi.it

**Keywords:** COVID-19, MDSC, platelet, L-Arginine

## Abstract

Massive platelet activation and thrombotic events characterize severe COVID-19, highlighting their critical role in SARS-CoV-2-induced immunopathology. Since there is a well-described expansion of myeloid-derived suppressor cells (MDSC) in severe COVID-19, we evaluated their possible role in platelet activation during SARS-CoV-2 infection. During COVID-19, a lower plasmatic L-arginine level was observed compared to healthy donors, which correlated with MDSC frequency. Additionally, activated GPIIb/IIIa complex (PAC-1) expression was higher on platelets from severe COVID-19 patients compared to healthy controls and inversely correlated with L-arginine plasmatic concentration. Notably, MDSC were able to induce PAC-1 expression in vitro by reducing L-arginine concentration, indicating a direct role of PMN-MDSC in platelet activation. Accordingly, we found a positive correlation between ex vivo platelet PAC-1 expression and PMN-MDSC frequency. Overall, our data demonstrate the involvement of PMN-MDSC in triggering platelet activation during COVID-19, highlighting a novel role of MDSC in driving COVID-19 pathogenesis.

## 1. Introduction

The ongoing COVID-19 pandemic due to the coronavirus SARS-CoV-2 remains a global health emergency. The clinical features of COVID-19 range from asymptomatic to severe pneumonia and fulminant disease [1], but the mechanisms responsible for this wide clinical presentation are not completely clear. In severe COVID-19, coagulation abnormalities appear, inducing a hypercoagulable state and an increased rate of thrombotic and thromboembolic events [2].

The high inflammatory response may contribute to the thrombotic complications by impairing procoagulant–anticoagulant balance, thus facilitating the development of microthrombosis and disseminated intravascular coagulation [3]. Further, the expression of SARS-CoV-2 receptor (angiotensin converting enzyme 2, ACE-2) on platelet membranes suggests a possible direct role of SARS-CoV-2 in platelet activation [4].

It has been shown that arginase I (Arg I) and nitric oxide synthase (iNOS) detract the microenvironment from arginine, inducing platelet activation, and impairing nitric oxide synthesis [5]. The myeloid derived suppressor cells (MDSCs) population is one of the main producers of ArgI and iNOS [6]; they strongly expand early after SARS-CoV-2 infection and can predict the fatal outcome of the disease [7,8]. MDSCs, defined in humans as HLADR low/- CD11b+ CD14- CD33+ CD15+ (polymorphonuclear, PMN-MDSCs) or HLA-DR low/- CD11b+ CD14+ CD33+ (monocytic, M-MDSCs) are known to have the remarkable ability to reduce inflammation by suppressing innate and adaptive immune function through several mechanisms, including iNOS, Arg-1, nicotinamide adenine dinucleotide phosphate oxidase (NOX2), and transforming growth factor beta (TGF-β) [9].

In this study, we assessed the capability of PMN-MDSC to activate platelets during SARS-CoV-2 infection. Our results showed a novel role of PMN-MDSC from COVID-19 patients, being able to increase platelet activation by reducing L-arginine concentration, thus contributing to the platelet hyperactivity observed in severe COVID-19.

## 2. Materials and Methods

### 2.1. Study Population

SARS-CoV-2 infected patients (*n* = 62) were treated at the National Institute for Infectious Diseases “Lazzaro Spallanzani” (Rome, Italy). We enrolled SARS-CoV-2 positive patients without other infections such as HIV, HCV, HBV, MTB, and others. Pregnant women were also excluded. All patients were symptomatic, ranging from moderate (PO2/FIO2 > 200, *n* = 31, no ICU) to severe (*n* = 31, requiring intensive care unit admission, ICU). Median age was 65 years (range 22–95), and 60% were males. ICU and no ICU patients (63.3% and 70%, respectively) presented one or more co-morbidities. These included hypertension (ICU = 53.3%, no ICU = 36.7%), cardiovascular diseases (ICU = 26.7%, no ICU = 10%), obesity (ICU = 26.7%, no ICU = 23.3%), diabetes (ICU = 16.7%, no ICU = 10%), and cancer (ICU = 10%, no ICU = 16.7). Healthy individuals (HD, *n* = 9) were included as controls.

The study was approved by the institutional review board (approval number: 9/2020) and signed written informed consent was obtained from patients.

### 2.2. Plasma Samples Preparation

Heparin anti-coagulated whole blood samples were centrifuged at 100× *g* for 15 min and platelet rich plasma (PRP) was collected for further use.

### 2.3. PBMC and PMN-MDSC Isolation

Peripheral blood mononuclear cells (PBMC) were isolated from heparin-treated whole blood by density gradient centrifugation (Lympholyte-H, Cederlane, Burlington, ON, USA) PBMC were suspended in RPMI 1640 (Corning Incorporated, NewYork, NJ, USA) and supplemented with 10% heat-inactivated fetal bovine serum (FBS) (EuroClone, Milan, Italy), penicillin/streptomycin solutions, and 2 mmol/L L-glutamine (Corning Incorporated, New York, NJ, USA).

PMN-MDSCs were isolated by using CD15 microbeads (MiltenyiBiotec, Bergisch Gladbach, Germany) according to the manufacturer’s procedure. Purity was >90% as verified by flow-cytometry (data not shown).

### 2.4. Platelets-PMN-MDSC Culture

Purified PMN-MDSCs (2 × 10^5^) were seeded in 96-well plate (Corning-Incorporated, New York, NJ, USA) in the above described medium without FBS. Twenty microliters of PRP from HD were added and cultured at 37 °C. After 4 h, platelet activation was evaluated by flowcytometry, and supernatants were collected for L-Arginine quantification.

### 2.5. Flow Cytometry

Platelet activation was analyzed by using anti-human REAfinity CD41 APC and anti-human REAfinity activated GPIIb/IIIa complex (PAC-1 PE mAb, Bergisch Gladbach, Miltenyi Biotec) on ice in the dark. After 15 min, 1% paraformaldehyde was added and samples were acquired by Cytoflex Flow Cytometer (Beckman-Coulter, Brea, CA, USA).

MDSC frequency was evaluated by staining PBMC with customized Duraclon tubes, (FITC-CD11b, ECD-HLA-DR, PC5.5-CD14, PC7-CD33, KrO-CD45, APC-CD80, DRAQ7, APC-alexa750-CD56, APC-alexa750-CD19, APC-alexa750-CD3, Pacific-Blue-CD15, and Beckman-Coulter) following manufacturer’s procedures. Data were acquired by CytoFlex flow-cytometer and analyzed by CytExpert (Beckman-Coulter, Brea, CA, USA).

### 2.6. L-arginine Quantification

Plasma samples and co-culture supernatants were centrifuged at 2000 rpm for 10 min to eliminate platelets and debris. L-arginine level was evaluated by UPLC-MS/MS by using Kairos Amino Acid Kit (Waters, Milford, MA, USA) according to the manufacturer’s instruction. Chromatographic separation was performed using an ACQUITY-UPLC system, followed by detection on a Xevo-TQD (Waters, Milford, MA, USA).

### 2.7. Statistical Analysis

GraphPad Prism version 8.00 (GraphPad Software) was used to perform statistical analyses. The non-parametric Kruskal-Wallis with Dunn’s post hoc test or the Wilcoxon matched-pairs signed rank test were used. Correlations were evaluated with the non-parametric Spearman test. The *p* < 0.05 was considered significant.

## 3. Results

### 3.1. Plasmatic L-Arginine in COVID-19 Patients was Correlated to PMN-MDSC Frequency

**Figure 1:**
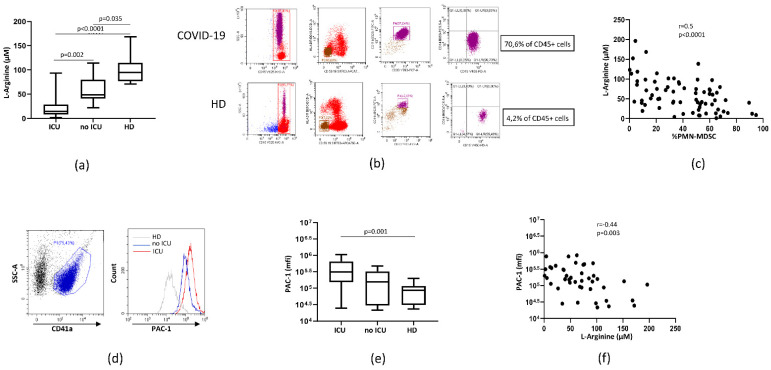
Plasmatic L-arginine level in SARS-CoV-2 patients correlated with PMN-MDSC frequency. (**a**) Plasmatic Arginine level of SARSCoV-2 ICU (ICU, *n* = 31) and no ICU (no ICU, *n* = 31) patients and healthy donors (HD, *n* = 9). Results are shown as box and whiskers plot. Kruskal-Walliswith Dunn’s post hoc test was applied. (**b**) Flow-cytometry gating strategy used to identify MDSC among PBMC. Dead cells were excluded by selecting DRAQ7neg cells. One representative COVID-19 patient and one HD are shown. (**c**) Correlation between plasmatic L-arginine level and PMN-MDSC percentage from SARSCoV-2 infected patients and HD. Non-parametric Spearman correlation was applied. (**d**) Representative plots of the adopted gating strategy to evaluate platelet activation. Platelets were selected as CD41a+ (SSC/CD41a plot), and PAC-1 expression (mean fluorescence intensity, mfi) was evaluated. (**e**) PAC-1 expression (mfi) on platelets from ICU (*n* = 21), no ICU (*n* = 15) patients, and HD (*n* = 9). Results are shown as box and whiskers plot. Kruskal-Walliswith Dunn’s post hoc test was applied. (**f**) Correlation between PAC-1 platelet expression (mfi) and L-arginine plasmatic level.

We evaluated the plasmatic concentration of L-arginine in patients with moderate (no ICU) and severe (ICU) COVID-19 and HD. A lower plasmatic L-arginine level was observed in both patient groups compared to HD (Figure 1a). Moreover, L-arginine was lower in ICU compared to no ICU patients, suggesting possible association with disease severity. PMN-MDSC percentage was evaluated by flow-cytometry (Figure 1b). A strong negative correlation was found between L-arginine level and PMN-MDSC frequency (Figure 1c), indicating that PMN-MDSC may be involved in the plasmatic L-arginine deprivation during COVID-19. To assess the role of arginine level in driving platelet activation, the expression of PAC-1 on CD41+ platelets was analyzed in a subgroup of SARS-CoV-2 infected patients and HD by flow-cytometry. The representative plots in Fig 1D show PAC-1 expression on platelets from ICU, non ICU patients, and HD. Cumulative analysis shows that the expression of PAC-1 on platelets was higher in ICU patients compared to HD (Figure 1e). In no ICU patients, an intermediate PAC-1 level was observed (Figure 1e), suggesting a higher platelet activation state in more severe patients. The expression of PAC-1 on platelets directly correlated with plasmatic L-arginine (Figure 1f), indicating that arginine shortage may be involved in platelet activation observed in severe COVID-19.

### 3.2. PMN-MDSC Induced Platelet Activation by Reducing L-Arginine

**Figure 2:**
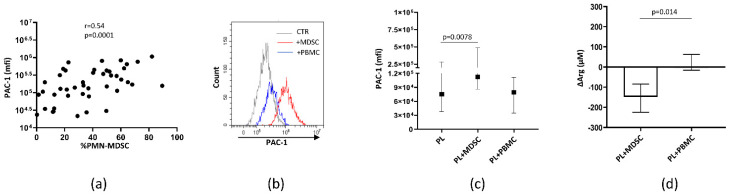
PMN-MDSC induced platelet activation by reducing L-arginine. (**a**) Correlation between PAC-1 platelet expression (mfi) and PMN-MDSC percentage from SARSCoV-2 infected patients (ICU *n* = 21, no ICU *n* = 15) and HD (*n* = 9). Non-parametric Spearman correlation was applied. (**b**) Representative histogram plot showing PAC-1 expression on platelets after culture with or without PMN-MDSC or PBMC depleted from MDSCs (PBMC). (**c**) PAC-1 expression on platelets from HD (*n* = 6) after culture with PMN-MDSC or PBMC from SARSCoV-2 infected patients (*n* = 6). Results are shown as median and IQR. Wilcoxon matched-pairs signed rank test was applied. (**d**) L-arginine reduction (ΔArg) calculated as the difference between L-arginine concentration in the presence of MDSCs or PBMC and platelets alone. Results are shown as median and IQR. A Wilcoxon matched-pairs signed rank test was applied.

Next, we evaluated whether PMN-MDSCs were directly involved in platelet activation. We found a positive correlation between PAC-1 platelet expression and PMN-MDSC frequency (Figure 2a), suggesting that PMN-MDSC may be involved in arginine deprivation. To confirm that PMN-MDSC may induce platelet activation, we cultured platelet rich plasma (PRP) from HD with PMN-MDSCs isolated from COVID-19 patients and, after 4 h, the expression of PAC-1 on platelets was evaluated by flow cytometry. We found that PMN-MDSCs from COVID-19 were able to activate resting healthy platelets by increasing the expression of PAC-1 (Figure 2b,c). As a control, PBMC depleted from MDSCs were used and no effect was observed. Accordingly, a reduction of L-arginine in the culture supernatants (Figure 2d) was found, indicating that PMN-MDSCs may activate platelets by depriving L-arginine from the microenvironment.

## 4. Discussion

Patients with severe COVID-19 commonly present thrombotic disorders, and these conditions have been associated with a higher mortality rate [10]. Moreover, severe COVID-19 is characterized by a strong neutrophilia that persists overtime. Among neutrophils, a strong inflammatory-driven expansion of PMN-MDSCs was observed in severe patients, which significantly reduces the adaptive immune response to SARS-CoV-2 and predicts a fatal clinical outcome [7,8].

In this paper, we analyzed an MDSC function never explored before, to the best of our knowledge, and showed that PMN-MDSCs from COVID-19 patients may be involved in platelet activation by reducing L-arginine availability, highlighting a new interplay between immune regulatory cells and platelet function.

According to previous published data [11], a decrease of L-arginine in the plasma from COVID-19 patients was found. In the present work, we also observed that L-arginine level inversely correlated with PMN-MDSC frequency and with platelet activation. Moreover, PMN-MDSC frequency directly correlated with platelet activation, suggesting a role of PMN-MDSCs in platelet activation during COVID-19. This hypothesis is corroborated by the high level of the enzymes involved in L-arginine catabolism, Arg I, and iNOS, expressed by PMN-MDSCs in COVID-19 patients [7]. Our in vitro experiments further provide a formal proof of the direct role of PMN-MDSC in inducing platelet activation through L-arginine consumption.

Platelet activation has been described during SARS-CoV-2 infection, and it is known to contribute to thromboembolic complications. Besides hyper-inflammation, factors such as a direct SARSCoV-2 infection and antibody-mediated mechanisms have been proposed to contribute to platelet hyperactivity [4]. In the present study, we demonstrated the PMN-MDSC as a new player in platelet homeostasis, highlighting an unprecedented function of the PMN-MDSC during COVID-19. Correlations between PMN-MDSC frequency with L-arginine concentration and with platelet activation have been shown during other infections such as severe fever caused by a bunyavirus [12]. Herein, we demonstrated that PMN-MDSC, by decreasing L-arginine, might directly contribute to platelet activation, shedding light on a novel role of PMN-MDSCs besides immune suppression.

## 5. Conclusions

Our findings demonstrate the direct involvement of PMN-MDSCs in platelet activation during COVID-19, confirming the MDSC expansion as one of the main events driving COVID-19 pathogenesis. These results also reveal new therapeutic perspectives targeting MDSC number and function, a promising strategy already under evaluation in cancer patients [13,14].

## Data Availability

Publicly available datasets were analyzed in this study. This data can be found here: [https://rawdata.inmi.it/].

## References

[1] Borges do Nascimento I.J., Cacic N., Abdulazeem H.M., von Groote T.C., Jayarajah U., Weerasekara I., Esfahani M.A., Civile V.T., Marusic A., Jeroncic A. (2020). Novel coronavirus infection (COVID-19) in humans: A scoping review and meta-analysis. J. Clin. Med..

[2] Bilaloglu S., Aphinyanaphongs Y., Jones S., Iturrate E., Hochman J., Berger J.S. (2020). Thrombosis in hospitalized patients with COVID-19 in a New York City health system. JAMA.

[3] Tang N., Li D., Wang X., Sun Z. (2020). Abnormal coagulation parameters are associated with poor prognosis in patients with novel coronavirus pneumonia. J. Thromb. Haemost..

[4] Shen S., Zhang J., Fang Y., Lu S., Wu J., Zheng X., Deng F. (2021). SARS-CoV-2 interacts with platelets and megakaryocytes via ACE2-independent mechanism. J. Hematol. Oncol..

[5] Pinto V.L., de Souza P.F., Brunini T.M., Oliveira M.B., Moss M.B., de Sá Siqueira M.A., Ferraz M.R., Mendes-Ribeiro A.C. (2012). Low plasma levels of L-arginine, impaired intraplatelet nitric oxide and platelet hyperaggregability: Implications for cardiovascular disease in depressive patients. J. Affect. Disord..

[6] Greten T.F., Manns M.P., Korangy F. (2011). Myeloid derived suppressor cells in human diseases. Int. Immunopharmacol..

[7] Sacchi A., Grassi G., Bordoni V., Lorenzini P., Cimini E., Casetti R., Tartaglia E., Marchioni L., Petrosillo N., Palmieri F. (2020). Early expansion of myeloid-derived suppressor cells inhibits SARS-CoV-2 specific T-cell response and may predict fatal COVID-19 outcome. Cell Death Dis..

[8] Falck-Jones S., Vangeti S., Yu M., Falck-Jones R., Cagigi A., Badolati I., Österberg B., Lautenbach M.J., Åhlberg E., Lin A. (2021). Functional monocytic myeloid-derived suppressor cells increase in blood but not airways and predict COVID-19 severity. J. Clin. Investig..

[9] Lee B.R., Chang S.Y., Hong E.H., Kwon B.-E., Kim H.M., Kim Y.-J., Lee J., Cho H.-J., Cheon J.-H., Ko H.-J. (2014). Elevated endoplasmic reticulum stress reinforced immunosuppression in the tumor microenvironment via myeloid-derived suppressor cells. Oncotarget.

[10] Levi M., Thachil J., Iba T., Levy J.H. (2020). Coagulation abnormalities and thrombosis in patients with COVID-19. Lancet Haematol..

[11] Reizine F., Lesouhaitier M., Gregoire M., Pinceaux K., Gacouin A., Maamar A., Painvin B., Camus C., Le Tulzo Y., Tattevin P. (2021). SARS-CoV-2-induced ARDS associates with MDSC expansion, lymphocyte dysfunction, and arginine shortage. J. Clin. Immunol..

[12] Li X.K., Lu Q.B., Chen W.W., Xu W., Liu R., Zhang S.-F., Du J., Li H., Yao K., Zhai D. (2018). Arginine deficiency is involved in thrombocytopenia and immunosuppression in severe fever with thrombocytopenia syndrome. Sci. Transl. Med..

[13] Eriksson E., Wenthe J., Irenaeus S., Loskog A., Ullenhag G. (2016). Gemcitabine reduces MDSCs, tregs and TGFbeta-1 while restoring the teff/treg ratio in patients with pancreatic cancer. J. Transl. Med..

[14] Tobin R.P., Jordan K.R., Robinson W.A., Davis D., Borges V.F., Gonzalez R., Lewis K.D., McCarter M.D. (2018). Targeting myeloid-derived suppressor cells using all-trans retinoic acid in melanoma patients treated with Ipilimumab. Int. Immunopharmacol..

